# Vorinostat suppresses hypoxia signaling by modulating nuclear translocation of hypoxia inducible factor 1 alpha

**DOI:** 10.18632/oncotarget.18125

**Published:** 2017-05-23

**Authors:** Chao Zhang, Chunzhang Yang, Michael J. Feldman, Herui Wang, Ying Pang, Dominic M. Maggio, Dongwang Zhu, Cody L. Nesvick, Pauline Dmitriev, Petra Bullova, Prashant Chittiboina, Roscoe O. Brady, Karel Pacak, Zhengping Zhuang

**Affiliations:** ^1^ Department of Orthopedics, Xinqiao Hospital, The Third Military Medical University, Chongqing, China; ^2^ Program in Reproductive and Adult Endocrinology, Eunice Kennedy Shriver National Institute of Child Health and Human Development, Bethesda, Maryland, USA; ^3^ Neuro-Oncology Branch, Center for Cancer Research, National Cancer Institute, Bethesda, Maryland, USA; ^4^ Surgical Neurology Branch, National Institute of Neurological Disorders and Stroke, National Institutes of Health, Bethesda, Maryland, USA; ^5^ Department of Molecular Medicine, Institute of Virology, Slovak Academy of Sciences, Bratislava, Slovakia

**Keywords:** HDACi, Hsp90, SAHA, HIF, hypoxia

## Abstract

Histone deacetylase inhibitors (HDACis) are a potent class of tumor-suppressive agents traditionally believed to exert their effects through loosening tightly-wound chromatin resulting in de-inhibition of various tumor suppressive genes. Recent literature however has shown altered intratumoral hypoxia signaling with HDACi administration not attributable to changes in chromatin structure. We sought to determine the precise mechanism of HDACi-mediated hypoxia signaling attenuation using vorinostat (SAHA), an FDA-approved class I/IIb/IV HDACi. Through an *in-vitro* and *in-vivo* approach utilizing cell lines for hepatocellular carcinoma (HCC), osteosarcoma (OS), and glioblastoma (GBM), we demonstrate that SAHA potently inhibits HIF-a nuclear translocation via direct acetylation of its associated chaperone, heat shock protein 90 (Hsp90). In the presence of SAHA we found elevated levels of acetyl-Hsp90, decreased interaction between acetyl-Hsp90 and HIF-a, decreased nuclear/cytoplasmic HIF-α expression, absent HIF-α association with its nuclear karyopharyin Importin, and markedly decreased HIF-a transcriptional activity. These changes were associated with downregulation of downstream hypoxia molecules such as endothelin 1, erythropoietin, glucose transporter 1, and vascular endothelial growth factor. Findings were replicated in an *in-vivo* Hep3B HRE-Luc expressing xenograft, and were associated with significant decreases in xenograft tumor size. Altogether, this study highlights a novel mechanism of action of an important class of chemotherapeutic.

## INTRODUCTION

Over the last decade the field of targeted chemotherapy has yielded significant improvements in the prognosis of patients with incurable malignancies. Despite the enthusiasm, there still exists a need for an effective therapy that interferes with components of the hypoxia signaling pathway. Transcriptional activity induced by the heterodimeric transcription factors hypoxia-inducible factor 1α and 2α (HIF-1α, HIF-2α) encourages neoplastic transformation to an aggressive phenotype through upregulation of genes involved with angiogenesis, tissue invasion, and metastasis [1–3]. Resulting intracellular metabolic changes facilitate tolerance to elevated levels of reactive oxygen species typically encountered after chemo- and/or radiotherapy [4–7]. Elevated intratumoral HIF levels carry a negative prognosis, and are an independent risk factor for decreased patient survival [8–10]. To this end, a variety of therapies inhibiting HIF and/or components of the hypoxia signaling cascade have been identified [11, 12]. However, agents that only target specific components of the hypoxia signaling pathway generally fail to produce an enduring clinical response [13–16]. The vast majority of HIF inhibitors utilized for pre-clinical and clinical investigation are non-specific for the hypoxia pathway [11].

Histone deacetylase inhibitors (HDACis) are a group of small-molecule compounds that have shown potent tumor-suppressive activity both in vitro and in clinical studies [17–21]. Their success led to FDA approval of two agents, Vorinostat (Suberoylanilide Hydroxamic Acid (SAHA), and Romidepsin (FK228), for treatment of cutaneous T cell lymphoma (CTCL) [22, 23]. There are currently over 120 clinical studies evaluating HDACi efficacy in other tumor subtypes [24]. The effects of HDACis are traditionally described as mediated through de-inhibition of genetic repression through loosening tightly wound chromatin [25–27]. HDACis prevent the removal of acetyl groups from the lysine residue on histone proteins, keeping the negatively charged acetyl molecule in close proximity to the negatively charged DNA phosphate backbone [27]. The mutual repulsion allows transcriptional access to genes generally suppressed by malignant cells, including tumor suppressors such as p21 and Rb [28–31]. Several recent studies have modified this view, clarifying class I HDACs (HDAC 1, 2, 3, 8) as the main class capable of acting on histones as their primary substrate [24]. Class I HDACs have shown the ability to promote cell proliferation and survival [29, 30], as well as endothelial sprouting and vascular branching [32]. However, it is the other classes of HDACs held largely accountable for angiogenesis initiation and propagation largely through the modification of non-histone proteins [32–40].

Knockout studies have demonstrated the vital importance of class IIa HDACs (HDAC4, 5, 7, 9), in particular HDAC7, for the development of the immature vasculature through cytoplasmic control of transcription factors [41]. Class IIb HDACs (HDAC6, 10) can act through HDAC6 to permit cytoplasmic accumulation of HIF through deacetylation of Hsp90 and ubiquitin [42]. Class III HDACs (SIRT1-7) act through Sirtuin 1 to deacetylate Foxo1, a transcription factor critical to blood vessel development [43–46]. Class IV HDACs consist solely of HDAC11, which has no known direct anti-angiogenic functions, rather functioning to regulate the immune system through control of cytokine expression [47, 48].

SAHA is a class I/IIb/IV HDACi that has potent inhibitory effects on the hypoxia signaling pathway. It's mechanism of hypoxia attenuation involves a high level of complexity due at least in part to its influence on multiple HDACs. SAHA has been shown to decrease levels of HIF-1α and VEGF in various tumor cell lines without a proportional change in the levels of HIF-1α mRNA [17, 32, 34]. Some authors have suggested this due to class II activity inducing direct HIFα acetylation, targeting HIF for destruction in a von Hippel-Lindau ubiquitin ligase (pVHL) or p53-dependent manner [38, 49, 50]. However, mechanistic studies have not reliably observed direct HIF-1α acetylation by N-acetyltransferase, and confirmatory studies have not seen increased pVHL activity after SAHA on a consistent basis [51–53]. Other groups have proposed a class I/II HDACi-mediated interaction with the Hsp70/90 chaperone axis causing increased ubiquitin-independent proteosomal degradation [54]. Even others suggest HIF translational inhibition through indirect interference with eukaryotic initiation factor-G3 (eIFG3) [34]. Clearly, a tremendous amount of interest has yielded multiple hypotheses for this complex mechanism. More evidence is needed to better understand SAHA-mediated hypoxia signaling suppression.

In the present study, we provide evidence suggesting that SAHA interferes with HIF signaling through direct acetylation of its associated chaperone, heat shock protein 90 (Hsp90). We propose this causes decreased HIF nuclear translocation mediated through decreased interaction of HIF with its nuclear karyopherin protein, Importin. Our findings were replicated across multiple tumor cells lines, and recapitulated in an *in-vivo* Hep3B HRE-Luc expressing xenograft. These data provide insight into the mechanism of action of the FDA-approved HDACi, SAHA, as well as identify Importin as a potential therapeutic target for treatment of hepatocellular carcinoma, and possibly other cancer sub-types characterized by aggressive hypoxia signaling.

## RESULTS

### Effects of SAHA on HIF-1 response to hypoxia

**Figure 1:**
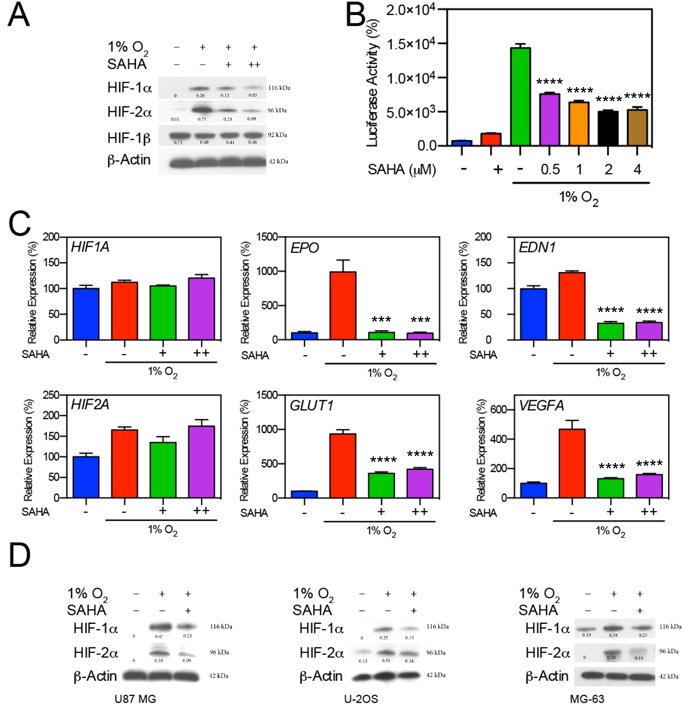
SAHA suppresses HIF-1α and HIF-2α induction in response to hypoxia **A.** Hep3B cells were exposed to hypoxic conditions (1% O_2_) for 16 hr in the presence of 0.5 or 1 μM of SAHA (+, and ++, respectively). Representative western blot with normalized densitometric values (protein/actin loading control) show decreases in HIF-1α and HIF-2α expression upon exposure to SAHA. **B.** Luciferase reporter assay demonstrates significant decreases (*p* < 0.0001) in HRE-associated luciferase activity in response to SAHA under hypoxic conditions. **C.** Effects of SAHA on hypoxia related gene expression in Hep3B cells exposed to SAHA for 16 hr under conditions of 21% or 1% O_2_ analyzed by qRT-PCR, showing significant suppression of *EDN1*, *EPO*, *GLUT1*, and *VEGF*A in response to both 0.5 and 1 μM SAHA with no significant change in *HIF1A* or *HIF2A* expression. **D.** Tumor cell lines U87 MG, U2OS, and MG63 were exposed to 0.5 μM SAHA for 16 hr under 21% or 1% O_2_, with resulting HIF-1α and HIF-2α suppression similar to that observed in Hep3B cells. Representative western blot with normalized densitometric values (protein/actin loading control) are shown. *p < 0.05, **p < 0.01, ***p < 0.001, ****p < 0.0001.

The effect of the type I/IIb/IV HDAC inhibitor SAHA on HIF expression was determined in multiple tumor-derived cell lines. In the hepatocellular carcinoma Hep3B cell line, exposure to low-dose (0.5 μM) and moderate-dose SAHA (1 μM) caused a decrease in the quantity of HIF-1α and HIF-2α under hypoxic conditions (Figure 1A). We evaluated the effect of SAHA on HIF-1/2α-associated transcriptional activation through a luciferase assay based on Hep3B cells carrying a stably transfected Hypoxia Responsive Element (HRE) luciferase reporter (Hep3B HRE-Luc). SAHA significantly reduced the transcriptional activity of HIF-1/2α under hypoxic conditions (*p* < 0.0001), while exerting minimal effect on HRE transcription under normal oxygen conditions (Figure 1B). In accordance with the reduction in the amount of HIF-1/2α HRE-reporter signaling, SAHA resulted in significant inhibition of hypoxia-responsive gene expression with downregulation of endothelin 1 (*EDN1*) (*p* < 0.0001), erythropoietin (*EPO*) (*p* < 0.001), glucose transporter 1 (*GLUT1*) (*p* < 0.0001), and vascular endothelial growth factor (*VEGFA*) (*p* < 0.0001). No significant change was observed in the mRNA expression levels of HIF1A or HIF2A in the same assay (Figure 1C). We confirmed the inhibitory effect of SAHA on the expression of HIF-1α and HIF-2α in glioblastoma (U87 MG) and osteosarcoma (U2OS and MG-63). The reduction of HIF-1/2α expression was similar among the conditions of low oxygen levels (1% O_2_) (Figure 1D), and in the presence of the hypoxia mimetic CoCl_2_ and oxoglutarate analog dimethyloxalylglycine (data not shown). This data suggests that SAHA suppresses HIF downstream transcriptional activity independent of a reduction in HIF mRNA levels.

### SAHA interferes with HIF-1/2α nuclear localization

**Figure 2:**
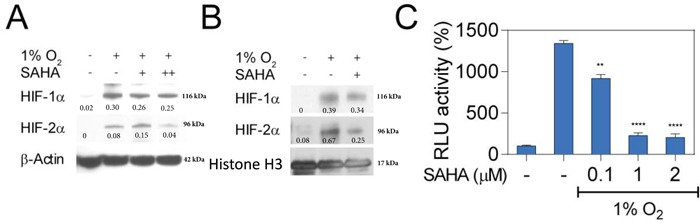
SAHA interferes with nuclear translocation of HIF-1α and HIF-2α **A.** Hep3B cells were cultured under normoxic or hypoxic conditions for 16 hr. Representative western blot with normalized densitometric values (protein/actin loading control) show no change in total cellular HIF-1/2α expression at low concentrations of SAHA (0.1 μM, +), and moderate decrease at 2 μM (++). **B.** Representative western blot with normalized densitometric values (protein/histone loading control) show nuclear proteins isolated from Hep3B cells exposed to 0.1 μM (+) SAHA for 16 hr under indicated conditions with decreased nucleated HIF-1α and HIF-2α. **C.** Luciferase reporter assay demonstrates a significant decrease in HRE-associated luciferase activity in response to SAHA under hypoxic conditions. *p < 0.05, **p < 0.01, ***p < 0.001, ****p < 0.0001.

To further investigate the underlying mechanisms of SAHA-mediated repression of HIF activity, we assessed HIF subcellular localization after incubation with SAHA. Notably, very low-dose SAHA treatment (0.1 mM) resulted in a slight decrease in total cellular HIF-1α levels in Hep3B cells (Figure 2A). A larger decrease in HIF-1α and HIF-2α concentrations were noted in the nuclear compartment after SAHA treatment (Figure 2B). Changes in HIF-expression were confirmed by luciferase assay demonstrating a significant reduction in HIF-associated signaling at very-low, moderate, and high SAHA concentrations (*p* < 0.01, *p* < 0.0001, *p* < 0.0001, respectively) (Figure 2C). Taken together, these findings suggest a SAHA-mediated hypoxia signaling interference contributing to out of proportion decreases in HIF nuclear concentrations and signaling relative to total cellular HIF levels.

### Suppression of HIF by both HDACi and Hsp90 inhibition

**Figure 3:**
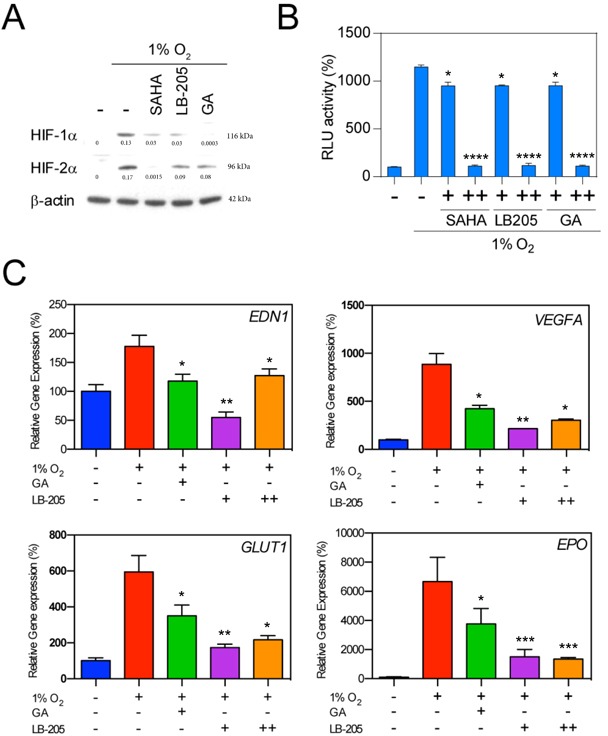
Effect of HDACi and Hsp90 inhibitor on HIFs expression **A.** Hep3B cells were exposed to the HDACis SAHA (1 μM), LB205 (2 μM), and the Hsp90 inhibitor geldanamycin (GA, 2 μM) under hypoxic culture conditions. Representative western blot with normalized densitometric values (protein/actin loading control) demonstrate a decrease in HIF-1α and HIF-2α levels in all treatment conditions. **B.** Luciferase reporter assay demonstrates a significant decrease in HRE-associated luciferase activity in response to 0.1 and 2 μM SAHA (+ and ++, respectively), 1 and 5 μM LB-205 (+ and ++, respectively), and 1 μM and 2 μM geldanamycin (+ and ++, respectively). **C.** Hypoxia related genes were suppressed by 2 μM GA, and with 1 and 5 μM of LB-205 (+ and ++, respectively). *p < 0.05, **p < 0.01, ***p < 0.001, ****p < 0.0001.

The broad spectrum class I/IIb/IV HDAC inhibitor LB-205 [55, 56], and the Hsp90 inhibitor geldanamycin (GA) were tested and compared to SAHA in the setting of hypoxia to determine whether SAHA leads to HIF downregulation at least partly through inactivation of Hsp90, and/or whether SAHA mediates HIF downregulation in a manner conserved across class I/IIb/IV HDAC inhibitors. We found that SAHA, LB-205, and GA each showed varying but elevated suppression of HIF-1α and HIF-2α (Figure 3A). Each agent was also associated with a significant reduction in HIF-α-associated transcriptional activity (Figure 3B). The inhibitory HIF-signaling effects of LB-205 and GA were further evidenced by qRT-PCR demonstrating a significant suppression of downstream hypoxia signaling transcripts EDN1, VEGFA, GLUT1, and EPO (Figure 3C). The observed downstream inhibition was comparable to that seen after SAHA exposure (Figure 1C).

### SAHA decreases HIF-Hsp90 binding affinity

**Figure 4:**
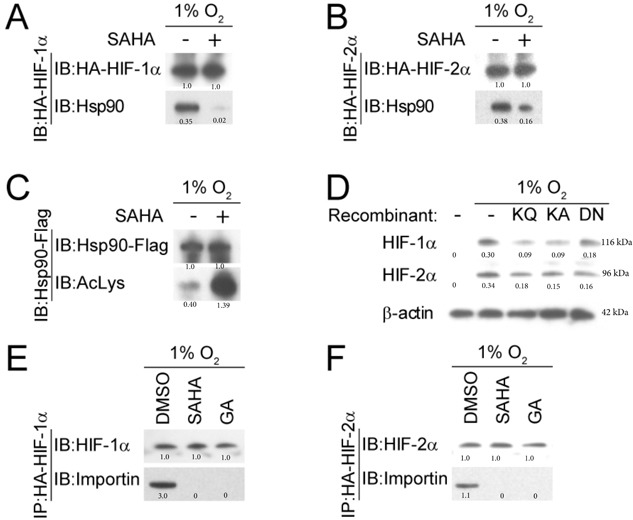
SAHA reduces the interaction between HIF-1/2α and Hsp90 **A.**, **B.** Representative western blot with normalized densitometric values (protein/HA-HIF loading control) after co-immunoprecipitation demonstrate diminished Hsp90 association with HIF-1α and HIF-2α in the presence of SAHA (1 μM). **C.** Hsp90 acetylation is increased by SAHA (1 μM), seen by immunoprecipitation with anti-Flag antibodies. Normalized densitometric values (protein/Hsp90-Flag loading control) are shown. **D.** Hsp90β recombinants that mimic acetylated (K286Q and K286A) and dominant-negative (D88N) forms of Hsp90β were cultured in Hep3B cells under hypoxic conditions, with resultant suppressed HIF-1α and HIF-2α expression compared to the WT Hsp90 (- recombinant) condition. Normalized densitometric values (protein/actin loading control) are shown. **E.**, **F.** Co-immunoprecipitation and representative western blot shows reduced HIF-α-importin interaction in the presence of SAHA (1 μM) or GA (1 μM). Normalized densitometric values (protein/HIF loading control) are shown.

Hsp90 is a chaperone that functions to assist protein folding and stabilization. Minet et al., demonstrated that Hsp90 interacts with HIF-1α in normoxic and hypoxic conditions, contributing to HIF-1α activation and turnover [57]. Hsp90 activity is modulated therapeutically by HDACis through acetylation of its middle domain lysine residue [58]. Acetylated Hsp90 likely exhibits less affinity to its client protein, making it less able to function in protein folding and other chaperone relevant functions. Targeting Hsp90 through inhibition of HDAC6 results in accumulation of acetylated Hsp90 [59, 60]. We demonstrated through co-immunoprecipitation assay that moderate-dose SAHA decreases the affinity between HIF-1/2α and Hsp90 (Figure 4A and 4B), with an associated concomitant increase in the amount of acetylated Hsp90 (Figure 4C). To assess whether Hsp90 acetylation affects the quantity of HIF-1α and HIF-2α, we measured HIF-1/2α expression under hypoxic conditions in the presence of the Hsp90b recombinants K286Q, K286A, and D88N. These recombinants have shown in prior experiments the ability to simulate the activity of acetylated Hsp90 [61, 62]. We found that the quantity of HIF-1α and HIF-2α was decreased in the presence of the Hsp90β recombinants (Figure 4D). An HRE-luciferase assay was performed that confirmed a significant reduction in HIF-α associated downstream activity in the recombinant groups (*p* < 0.0001) (Supplementary Figure S1). We investigated the effect of SAHA on HIF-α nuclear translocation by studying the interaction between HIF-α and Importin, a karyopherin located on the nuclear membrane that facilitates HIF's nuclear transport. We found that in hypoxic conditions, HIF-1α and HIF-2α associate with Importin in Hep3B cells (Figure 4E and 4F). In the presence of SAHA, no interaction occurs between the two proteins (Figure 4E and 4F). This suggests that SAHA-mediated acetylation of Hsp90 leads to an attenuation of HIF-1/2α interaction with Importin (Figure 4E and 4F). We repeated the experiment in the presence and absence of the Hsp90 inhibitor GA, and similarly found that Hsp90 inactivation led to absent interaction between HIF-1/2α and Importin (Figure 4E and 4F). Together, these findings suggest that SAHA-mediated acetylation of Hsp90 affects Hsp90 function, contributing to decreased Hsp90-binding with HIF, and decreased/absent HIF binding to Importin. Given the function of Importin as a nuclear karyopherin, this suggests that SAHA interferes with HIF signaling at least partly by interfering with HIF nuclear translocation.

### SAHA enhances HIF-1/2α ubiquitination and degradation

**Figure 5:**
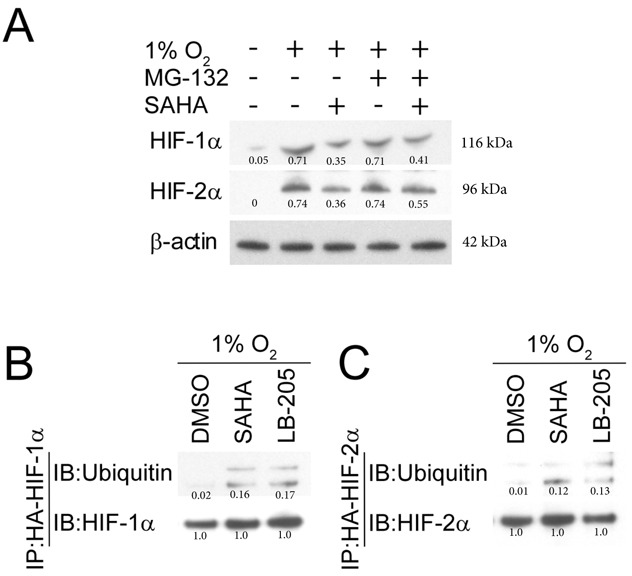
SAHA-induced degradation of HIF-1/2α is mediated by the ubiquitin and proteasome pathway **A.** The proteasome inhibitor MG132 (5 μM) reverses SAHA (1 μM) mediated HIF-1α and HIF-2α downregulation in Hep3B cells exposed to hypoxic conditions for 8 hours. Normalized densitometric values (protein/actin loading control) are shown. **B.**, **C.** Representative western blot with densitometric values after co-immunoprecipitation show exposure to SAHA (1 μM) or LB-205 (2 μM) resulting in increased HIF-ubiquitin association. Normalized densitometric values (protein/HIF loading control) are shown.

Under normoxic conditions, HIF-α is efficiently removed from the cytoplasm in a VHL-mediated E3 ubiquitin-ligase pathway involving the 26S proteasome [63]. We investigated HIF-1α and HIF-2α levels after giving SAHA in the presence/absence of MG-132, a 26S proteosome inhibitor that reverses pVHL ubiquitin-dependent degradation [63–67]. Consistent with our prior observations, the quantity of HIF-1/2α is reduced after moderate dose-SAHA therapy in hypoxic conditions. We found that HIF-1α and HIF-2α degradation was partly rescued by MG132, reaffirming the role of the 26S proteasome in SAHA-mediated HIF-1/2α degradation (Figure 5A). An immunoprecipitation assay was performed in the presence of SAHA to verify that SAHA increases HIF's association with ubiquitin, given ubiquitin's close functional role with the 26S proteasome, and given SAHA's ability to reverse 26S proteasome-mediated degradation of HIF. We found that HIF-ubiquitin association is enhanced in the presence of SAHA. To determine whether the observed effects were specific to SAHA or conserved across a similar class HDAC inhibitor, we replicated the experiment with LB-205. We found that LB-205 increases HIF ubiquitination to a similar degree as SAHA. These findings suggest that SAHA increases HIF degradation at least partly through a ubiquitin-dependent mechanism, in a manner likely conserved across I/IIb/IV HDAC inhibitors (Figure 5B-5C).

### SAHA reduces tumor burden and hypoxia signaling in tumor xenografts

**Figure 6:**
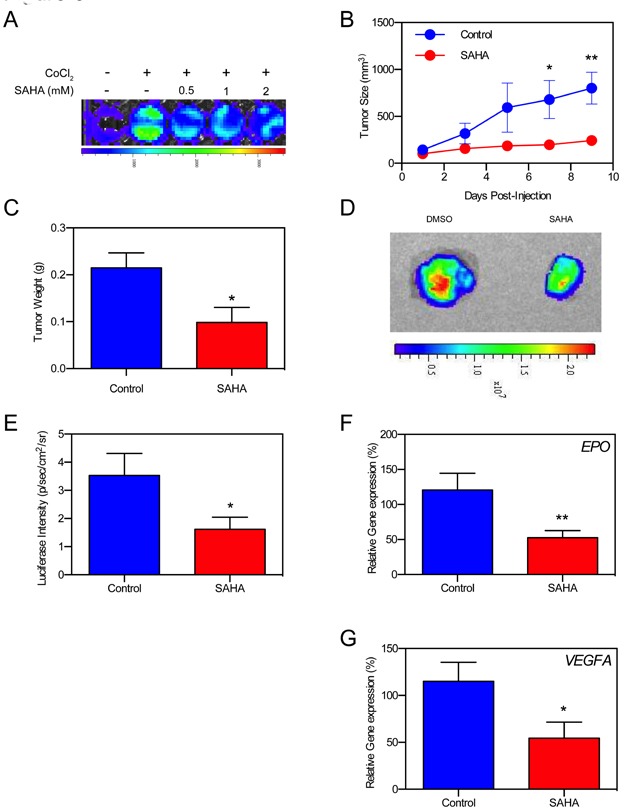
SAHA suppressed hypoxia signaling in Hep3B cell xenografts *in vivo* **A.** *In-vitro* luciferase assay shows decreased HRE activity in Hep3B HRE-Luc cells in the presence of SAHA. **B.** Tumor growth curves of Hep3B HRE-Luc cell xenografts treated with SAHA show significantly decreased tumor size at 7- and 9-days post-implantation (*p* < 0.05, *p* < 0.01, respectively). **C.** Average tumor weight of excised xenografts is significantly reduced in SAHA-treated mice compared to control mice (*p* < 0.05) at study endpoint. **D.** Example luminescence of excised Hep3B HRE-Luc cell xenografts at study endpoint. **E.** HRE-luc luminescence is significantly decreased in SAHA treated mice compared to control (*p* < 0.05) at study endpoint. **F.**, **G.** *VEGFA* and *EPO* expression is significantly decreased in tumors resected from SAHA treated mice at study endpoint. *p < 0.05, **p < 0.01, ***p < 0.001, ****p < 0.0001.

We used an HRE-Luc expressing Hep3B mouse xenograft model to validate SAHA's tumor-suppressive abilities against Hep3B, while also observing differences in real-time hypoxia signaling activity. Cells were validated *in-vitro* to report HIF-1/2α signal in pseudohypoxic (CoCl_2_) conditions (Figure 6A). Five million Hep3B HRE-Luc cells were subcutaneously injected in the right flank of SCID mice forming solid tumor masses within 10 days of implantation. Every other day (Q.O.D) administration of SAHA into the mouse peritoneum resulted in significant reductions of tumor volume and excision weight compared to non-treated controls (Figure 6B and 6C). We analyzed *in-vivo* hypoxia signaling activity at study endpoint, and found that mice receiving SAHA had diminished Hep3B HRE-Luc BLI signals (Figure 6D). Analysis of excised tumors confirmed that BLI signals originated from the tumors, with a significantly decreased average BLI vs control (*p* < 0.05) (Figure 6E). Intratumoral expression of the downstream hypoxia signaling mediators *EPO* and *VEGFA* were significantly decreased in Hep3B HRE-Luc xenografts (*p* < 0.01, *p* < 0.05, respectively) (Figure 6F and 6G). SAHA's tumor-suppressive effect was replicated in a pseudohypoxic 786-O renal cell carcinoma mouse xenograft model characterized by constitutive HIF signaling (even in the absence of hypoxia) due to absent pVHL protein, which is necessary for ubiquitin-dependent degradation of HIF [68, 69]. We found a significant reduction in 786-O tumor volume with SAHA therapy starting at day 8 post-injection, becoming more pronounced until the study endpoint (*p* < 0.05, *p* < 0.001, respectively) (Supplementary Figure S2).

**Figure 7:**
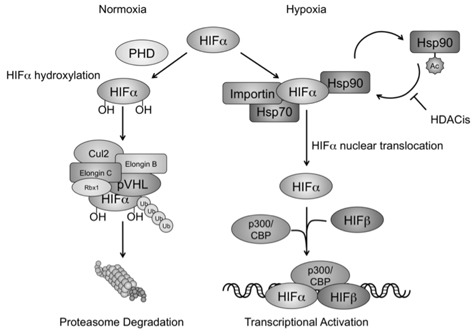
HDACis interfere with hypoxia signaling by affecting Hsp90 acetylation and HIF-α nuclear translocation In normoxic conditions, HIF-1/2α are first modified by prolyl hydroxylase (PHD) for protein hydroxylation, and removed via the VHL associated proteasomal degradation pathway. In hypoxic conditions, HIF-α recruits Hsp70 and Hsp90 in the cytoplasm, and interacts with the karyopherin importin for nuclear translocation. Nucleated HIF-α further recruits other cofactors such as HIF-b and p300/CBP, and initiates gene transcription and hypoxia signaling. HDACis act to increase accumulation of the acetylated form of Hsp90. The reduction of Hsp90 chaperone activity and HIF-a recognition results in HIF-α nuclear translocation and transcriptional activation.

## DISCUSSION

HDACis are an important class of chemotherapeutics that mediate much of their anti-neoplastic activity through suppression of the hypoxia signaling pathway. The FDA-approved class I/IIb/IV HDACi agent SAHA has shown efficacy in pre-cinical settings against a variety of cancer sub-types including pancreatic, breast, prostate, colon, and liver; as well as in clinical settings for the treatment of cutaneous T cell lymphoma [70]. However, its mechanism of HIF signaling suppression has yielded multiple hypotheses without a clear indication of relative influences. Much of the complexity results from different cell lines used across studies, and from results obtained from alternate-class HDACis grouped together with SAHA. We sought to clarify the mechanism of SAHA-mediated hypoxia suppression using a variety of different cell lines, as well as an *in-vivo* model capable of demonstrating real-time hypoxia signaling. While our findings support many of the conclusions obtained from several other studies, they also support a novel mechanism of SAHA-mediated hypoxia suppression. Our study demonstrates that SAHA disrupts Hsp90 function through direct Hsp90 acetylation, and contributes to decreased HIF-Hsp90 affinity, diminished HIF-Importin interaction, and decreased intranuclear HIF levels. These findings suggest a mechanism of SAHA-mediated hypoxia suppression involving interference with HIF's ability to translocate the nuclear membrane.

Studies have shown that elevated HIF-1 activity stimulates malignant transformation by promoting angiogenesis, epithelial-mesenchymal transition, invasion, metastasis, and resistance to traditional chemotherapy agents [1–3]. SAHA is implicated in interfering with HIF-1α signaling at the post-translational level through class I and class IIb HDAC inhibition [32, 42]. We found that SAHA administration at low- and moderate-doses is associated with decreased expression of HIF-1α and HIF-2α without a corresponding decrease in HIF-1α or HIF-2α mRNA across a variety of cell types (Figure 1A-1C). These findings support prior studies that demonstrated HIF-1α downregulation after administration of SAHA [17, 35, 38]. Kong attributed HIF-1α downregulation after SAHA administration to disruption of the Hsp70/90 chaperone axis [54]. We compared HIF levels after administration of SAHA, with administration of the Hsp90 inhibitor GA, and the Class I/IIb/IV HDACi LB-205. We observed a similar decrease in HIF-1α and HIF-2α expression in each treatment group, suggesting that SAHA-mediated downregulation of HIF is largely attributable to dysfunction of Hsp90. GA and LB-205 similarly decreased HRE-activity, and downstream hypoxia transcript expression (Figure 3).

Previous studies have shown that the class IIb HDAC6 directly acetylates the middle domain lysine residue of Hsp90, rendering it largely non-functional [58–60]. Our findings support moderate-dose SAHA leading to Hsp90 acetylation, Hsp90 dysfunction, and increased HIF degradation. We observed with exposure to SAHA a larger precipitant composed of Hsp90 and anti-acetylated lysine compared to hypoxia-only controls (Figure 4C), a smaller precipitant composed of HIF-1/2α and Hsp90 than hypoxia-only controls (Figure 4A-4B), and decreased HIF-1α and HIF-2α levels in cells with recombinant acetyl-Hsp90 mimetics relative to Hsp90-only controls (Figure 4D). WT Hsp90 Hep3B HRE-luc cells additionally demonstrated significantly increased HRE activity during hypoxic conditions than Hep3B HRE-luc cells with acetyl-Hsp90 recombinants in the presence of hypoxia (*p* < 0.0001) (Supplementary Figure S1).

Reports are mixed regarding the manner in which HIF-1α undergoes degradation in response to SAHA. Whereas Kong described SAHA-mediated HIF-1α degradation occuring in a ubiquitin-independent proteosomal manner [54], other groups proposed HIF degradation due to a ubiquitin-dependent proteosomal process resulting from direct HIF acetylation [38, 49, 50]. Our findings support SAHA-mediated HIF degradation occurring through increased HIF ubiquitination. We observed Hep3B cells exposed to hypoxic conditions+SAHA+MG132 to have elevated levels of HIF compared to hypoxia+SAHA only (Figure 5A). We also found an increased HIF-ubiquitin aggregate in Hep3B cells exposed SAHA+hypoxia relative to hypoxia-alone (Figure 5B-5C). SAHA-associated HIF degradation might be generalizable to class I/IIb/IV HDACis, as we found a similar degree of HIF-ubiquitin association with LB-205 as with SAHA.

Hsp90 was investigated in this study since it is a known target of SAHA that is heavily involved in HIF-1/2a signaling. SAHA decreases tumor neovascularization, causes abnormal tumor vascular morphology, and interferes with endothelial cell migration, proliferation, and survival at least partly due to decreased hypoxia signaling [71–74]. Pharmacologic inhibition of Hsp90 with Geldanamycin demonstrates similar *in-vitro* and *in-vivo* findings also through downregulation of the hypoxia signaling cascade [75–77]. Conditional knockout models of Hsp90 could provide another degree of validation regarding SAHA-mediated hypoxia signaling interference occurring through Hsp90 impairment. Conventional Hsp90 knockout models are generally non-viable in eukaryotic cells. The role of Hsp70 or the other Heat Shock proteins on HIF signaling after SAHA exposure could also be investigated. Hsp70 in particular has been implicated in altering HIF signaling after Hsp90 inactivation [78–80]. However, it not a direct target of SAHA, and likely plays an indirect role, if at all, during SAHA exposure [81]. Our study found absent HIF-Importin association after SAHA exposure (Figure 4E-4F), which was out of proportion to the degree of HIF downregulation seen after exposure to either hypoxia+acetyl-Hsp90 mimetics (Figure 4D) or hypoxia+SAHA-alone (Figure 2A-2B). If other HSP proteins are involved in permitting HIF signaling during SAHA-mediated Hsp90 inactivation, at least some HIF-Importin association would likely have been seen.

Future studies could perform reciprocal immunoprecipitation of Hsp90/HIF/Importin to determine whether they form a tertiary complex near the nuclear membrane. Currently, the molecular arrangement of these molecules is unknown. Our *in-vivo* HCC xenograft demonstrated significantly decreased HRE-luciferase intensity (*p* < 0.05) (Figure 6D-6E), and significantly less intratumoral EPO and VEGFA expression (*p* < 0.01 and *p* < 0.05, respectively) at the study endpoint (Figure 6E). Other studies could focus on this aspect and perform short-interval intratumoral RT-PCR of hypoxia signaling transcripts and/or imaging of HRE-luciferase activity to assess hypoxia signaling differences as the tumors grow in size. Real-time tumor oxygenation/perfusion could also be investigated using PtO_2_ polarographic electrodes, or via non-invasive imaging methods such as MRI-perfusion. MRI with contrast could possibly be used to identify whether changes in intratumoral vessel permeability occur over time.

In summary, we report that the HDAC inhibitor SAHA regulates hypoxia signaling by directly acetylating Hsp90 causing an increase in ubiquitin-mediated HIF degradation, and an attenuation of HIF-Importin association. *In-vitro* findings were recapitulated in an *in-vivo* model that demonstrated diminished hypoxia signaling in animals exposed to SAHA monotherapy. SAHA additionally exhibited potent *in-vivo* tumor suppressive activity against Hep3B and 786-O xenografts. We believe these findings highlight the role of Importin in SAHA-mediated hypoxia signaling suppression, and provide preclinical support for testing SAHA in clinical trials for HCC.

## MATERIALS AND METHODS

### Cell culture

Hep3B, HepG2, U2OS, MG63 and U87 MG cells were purchased from American Type Culture Collection (ATCC, Manassas, Virginia, USA) and cultured according to manufacturer's instructions. Hypoxia (1% O_2_, 5% CO_2_) conditions were established using an oxygen station (In VIVO2; Baker Ruskinn Tech, Stanford, Maine, USA). The Hep3B HRE-Luc cell line was established by infecting Hep3B cells with Cignal Lenti HIF Reporter (Luc) lentivirus (QIAGEN, Venlo, Limburg). Cells were selected in the presence of 5 mg/mL puromycin (Sigma, St. Louis, Missouri, USA). The luciferase signal was determined spectrophotometrically (Molecular Devices; Sunnyvale, CA).

### Quantitative PCR

Total RNA was extracted from cell pellets using the RNeasy Mini Kit (QIAGEN). Genomic DNA was removed through in-column DNaseI digestion (QIAGEN). cDNA was reverse-transcribed using Super Script III First-Strand Synthesis SuperMix (Invitrogen, Carlsbad, California, USA). The cDNA products were analyzed on an Eco Real-Time PCR System (Illumina, San Diego, California, USA). The primer sets used in the present study were: *EDN1* (Origene HP205717), *EPO* (Origene HP200740), *GLUT1* (Origene HP209446), *VEGFA* (QIAGEN QT01682072), and *ACTB* (Promega G5740). Data analysis was performed using Microsoft Excel and GraphPad Prism (version 6.0d, San Diego, California, USA).

### Western blot analysis

Cell pellets were harvested and lysed in RIPA lysis buffer supplemented with Complete Protease Inhibitor Cocktail (Roche, Basel, Switzerland) and 0.5% SDS. Protein was quantified using the Bio-Rad Protein Assay Kit (Bio-Rad, Berkeley, California). Samples were separated by NuPAGE Bis-Tris 4–12% gel (Invitrogen, Carlsbad California) and transferred onto PVDF membranes (Millipore, Billerica, Massachusetts, USA). The membranes were probed with primary antibody and detected through an HRP-conjugated species-specific secondary antibody and an ECL kit (Pierce, Waltham, Massachusetts, USA). The primary antibodies used in this study include: antibodies against HIF-1α [82–85], HIF-2α (Novus, Littleton, Colorado, USA), ubiquitin (Abcam, Cambridge, UK), Hsp90 (Cell Signaling Technology, Danvers, Massachusetts, USA), acetyl-lysine (Millipore, Billerica, Massachusetts, USA) and β-Actin (Santa Cruz Biotechnology, Santa Cru.z, California, USA). Antibodies were chosen based on existence of thorough external validation and widespread use in other well-respected peer-reviewed journals. Three independent experiments were performed for each representative immunoblot. Normalized densitometric values (protein/loading control) were calculated and displayed.

### DNA cloning and site-directed mutagenesis

Human HSP90AB1 gene was incorporated into the pCMV6-entry vector (Origene) as previously described [61, 62]. Mutagenesis of the K286 site was performed using the QuikChange Lightning Site-Directed Mutagenesis Kit (Agilent). The nucleotide sequence of the HSP90AB1 gene was verified by analyzing the entire coding regions through Sanger sequencing. The HSP90AB1 dominant negative vector (Hsp90β-D88N) was a gift from William Sessa (Department of Pharmacology and Vascular Biology and Therapeutics Program, Boyer Center for Molecular Medicine, Yale University School of Medicine, New Haven, CT).

### Immunoprecipitation

Immunoprecipitation was performed per manufacturer's instructions. Cell pellets were lysed in RIPA buffer with Protease Inhibitor Cocktail and 0.5% SDS. Total cell lysate was precipitated using the DynaBeads Protein G Immunoprecipitation Kit (Invitrogen) and antibodies against FLAG-tag (Origene, Rockville, Maryland, USA) or HA (Sigma-Aldrich, St. Louis, Missouri, USA). Precipitated protein was eluted and resolved by Western blot analysis. Three independent experiments were performed for each representative immunoblot provided in the figures. Normalized densitometric values (protein/loading control) were obtained and displayed.

### *In vivo* studies

Animal experiments were approved for use and care of animals under the guidelines of NIH animal protocol (1349-13). Six to eight-week-old female nude athymic mice (nu/nu) were obtained, with each mouse weighing approximately 20 grams at the onset of the experiment. Mice were injected subcutaneously in right flank with 5×10^6^ Hep3B HRE-Luc cells. After the xenografts reached 0.5 cm (day 1), animals were randomized to 2 groups of 3 animals each. Animals were treated intraperitoneally with vehicle alone or with SAHA at a dose of 5mg/kg on days 1, 3, 5, 7 and 9. Tumors were measured every other day using calipers. Tumor volumes were calculated from 2-dimensional measurements using the formula (tumor volume=length×width^2^). The animals were imaged at day 9 post-xenograft by bioluminescence imaging (BLI). Mice were anesthetized with isoflurane for imaging, and euthanized using CO_2_ inhalation or cervical dislocation. Tumors from the mice were imaged after excision, and expression profiles were assessed for HIFs downstream gene expression by qPCR using the above-described RT-PCR protocol.

## SUPPLEMENTARY MATERIALS FIGURES


