# Supplementary material for: Vorinostat suppresses hypoxia signaling by modulating nuclear translocation of hypoxia inducible factor 1 alpha
Source: Oncotarget. 2017 May 23;8(34):56110–25. doi: 10.18632/oncotarget.18125 (PMC5593548; doi:10.18632/oncotarget.18125)
Supplement: Supplementary file 1 [file oncotarget-08-56110-s001.pdf]

Vorinostat suppresses hypoxia signaling by modulating nuclear translocation of hypoxia inducible factor 1 alpha

SUPPLEMENTARY MATERIALS

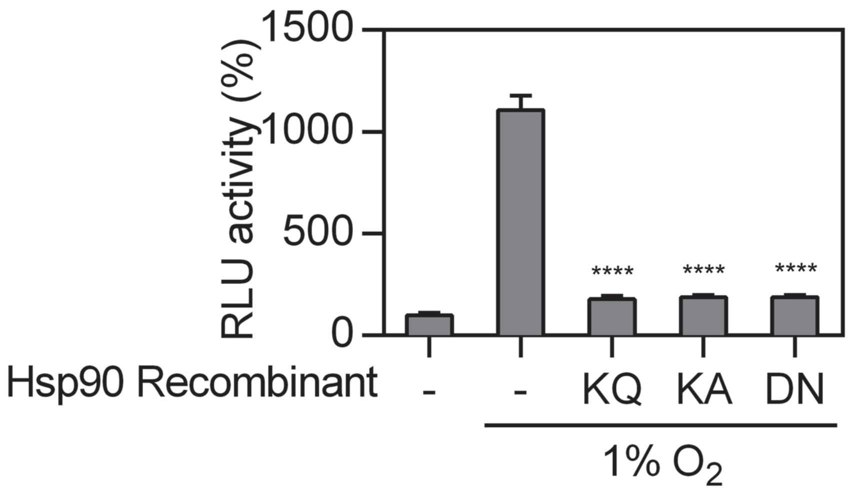

Supplementary Figure 1: Luciferase assay for Hsp90 acetylation mimetic recombinants. HRE-luciferase activity was significantly decreased with hypoxia + acetyl-Hsp90 mimetics: K286Q (KQ), K286A (KA), or dominant negative Hsp90 (D88N, DN) versus Hsp90 WT control (-) ($p<0.0001$).

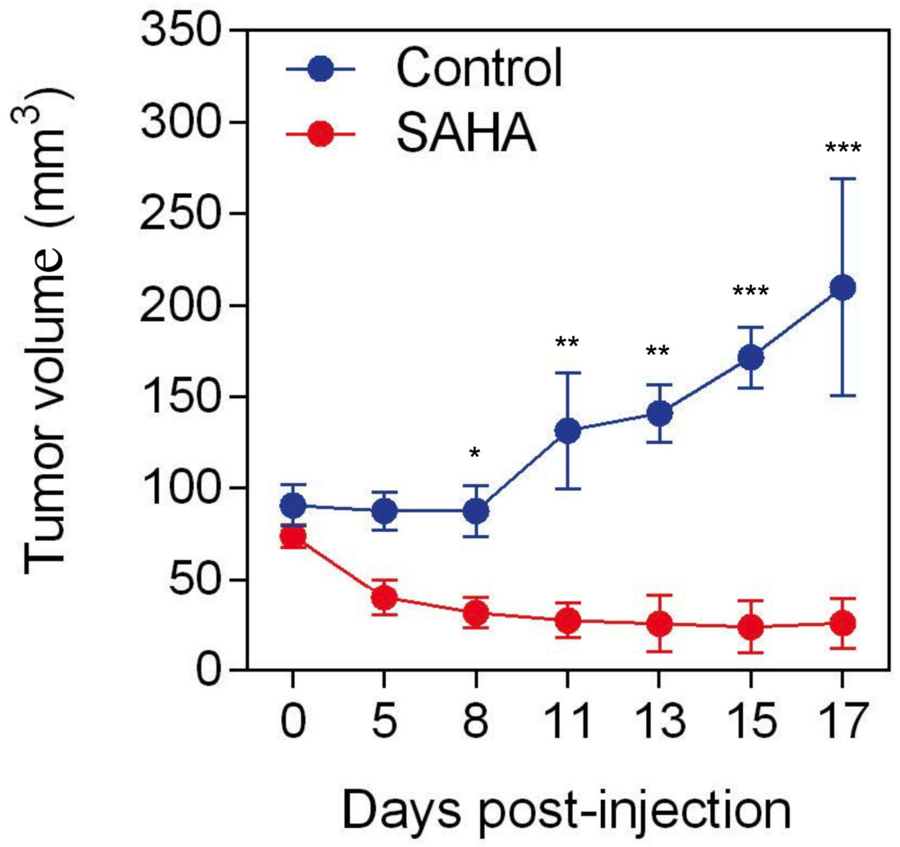

Supplementary Figure 2: SAHA reduced tumor growth in vivo. Subcutaneous xenografts were made by injection of 106 786-O cells were implanted in the right flank of SCID mice. SAHA treatment was started when the tumor volume reached 100 mm3. Tumor growth curves show a significantly suppressed tumor growth rate. *p<0.05, **p<0.01, ***p<0.001, ****p<0.0001.
